# Adult-born neurons maintain hippocampal cholinergic inputs and support working memory during aging

**DOI:** 10.21203/rs.3.rs-1851645/v1

**Published:** 2023-01-31

**Authors:** Alex Dranovsky, Greer Kirshenbaum, Chia-Yuan Chang, Maria Bompolaki, Victoria Bradford, Joseph Bell, Stylianos Kosmidis, Rebecca Shansky, Javier Orlandi, Lisa Savage, Eduardo Leonardo, Alexander Harris

**Affiliations:** Columbia University, New York State Psychiatric Institute; Columbia University, New York State Psychiatric Institute; Columbia University, New York State Psychiatric Institute; Columbia University, New York State Psychiatric Institute; Columbia University, New York State Psychiatric Institute; Columbia University, New York State Psychiatric Institute; Columbia University, New York State Psychiatric Institute; Mount Sinai School of Medicine; Columbia University, New York State Psychiatric Institute; Columbia University; Columbia University

## Abstract

Adult neurogenesis is reduced during aging and impaired in disorders of stress, memory, and cognition though its normal function remains unclear. Moreover, a systems level understanding of how a small number of young hippocampal neurons could dramatically influence brain function is lacking. We examined whether adult neurogenesis sustains hippocampal connections cumulatively across the life span. Long-term suppression of neurogenesis as occurs during stress and aging resulted in an accelerated decline in hippocampal acetylcholine signaling and a slow and progressing emergence of profound working memory deficits. These deficits were accompanied by compensatory reorganization of cholinergic dentate gyrus inputs with increased cholinergic innervation to the ventral hippocampus and recruitment of ventrally projecting neurons by the dorsal projection. While increased cholinergic innervation was dysfunctional and corresponded to overall decreases in cholinergic levels and signaling, it could be recruited to correct the resulting memory dysfunction even in old animals. Our study demonstrates that hippocampal neurogenesis supports memory by maintaining the septohippocampal cholinergic circuit across the lifespan. It also provides a systems level explanation for the progressive nature of memory deterioration during normal and pathological aging and indicates that the brain connectome is malleable by experience.

## Introduction

Connectivity between brain systems is thought to be established during developmental critical periods and then remain relatively fixed in adults. However, neurogenesis persists in the hippocampus throughout life, raising a possibility that the addition of new cells could impact the stability of long-range connections over time. This is especially intriguing since adult neurogenesis is highly susceptible to the effects of environmental changes, neuropsychiatric diseases, and aging [1-3], presenting a putative mechanism for a very slow form of experience-based circuit plasticity in the adult brain. Stress and aging-induced reductions in neurogenesis correlate with decreased cognitive [4] and emotional [5] flexibility. However, little is known about how ongoing neurogenesis influences hippocampal circuitry and function over time.

The importance of young neurons in hippocampal functions has been closely examined. Several studies have elucidated the dynamic properties of new hippocampal neurons throughout their maturation, and others have illuminated how new neurons integrate into existing brain circuits [6]. Young (4–12-week-old) hippocampal neurons have been found to play a role in the more difficult versions of hippocampal dependent cognitive tasks [7, 8] and a subtle, but significant role in stress regulation [5]. These findings emerged from short-term experimental reductions or increases in neurogenesis in rodents and together indicate an important contribution of young neurons to hippocampal function. However, neurogenesis is an ongoing process, raising the possibility that long-term changes in the addition of new neurons to existing circuits could result in system-wide changes with more dramatic consequences for hippocampal function and for behavior. This notion is especially intriguing since it encompasses time scales that reflect aging and the chronicity of illnesses where neurogenesis and hippocampal function are impaired. We therefore hypothesized that long periods of reduced neurogenesis, as observed in aging and chronic stress, would influence hippocampal connectivity with other brain systems. Since new neurons are added throughout life, we expected that long periods of reduced neurogenesis could lead to substantial connectivity changes, which would be associated with circuit dysfunction with behavioral consequences.

We investigated whether long term reductions in hippocampal neurogenesis influence inputs into the hilus where hippocampal neurogenesis occurs. We permanently reduced neurogenesis in adult mice and observed a slowly progressing impairment of hippocampal acetylcholine signaling that ultimately led to a progressive decline in working memory. These changes corresponded to a profound remodeling of the cholinergic septohippocampal projection with increased dysfunctional cholinergic innervation to the ventral hippocampus and recruitment of ventrally projecting neurons for innervation of the dorsal hilus. Selective expression of activating DREADDS in cholinergic neurons rescued working memory deficits in animals devoid of neurogenesis and in those with age-related cognitive decline.

## Materials And Methods

### Animals

Male C57BL/6J mice or ChAT-Cre animals (JaxMice) were used for X-irradiation studies as described previously [9]. Mice were delivered to our animal facility at 7-weeks-old and acclimated for one week before sham or X-irradiation treatment at 8 weeks. ChAT-Cre animals were maintained as heterozygotes on a C57Bl6J background.

GFAP-Tk heterozygous mice (NG−^TK^; [10]) express herpes thymidine kinase (Tk) under control of the glial fibrillary acidic protein (GFAP) promoter. Treatment with Valgancyclovir (VGCV) in NG−^TK^ mice leads to reductions in cell proliferation in dividing stem cells with relative sparing of non-stem astrocytes [11]. Female GFAP-Tk heterozygous mice were mated with wild-type littermate males, all on C57BL/6J 129S6 mixed background. Male pups were genotyped using PCR as described [10], weaned at P21, and housed 3–5 per cage with mixed genotypes. Half the mice were GFAP-Tk heterozygous (NG−^TK^) and half were negative for the gene (NG + ^Tk^). At 8 weeks mice were started on a feeding schedule of chow containing VGCV as described below.

Mice were given *ad libitum* access to food and water under a 12:12 h light:dark cycle in a temperature-controlled (72°F) colony. All animal experiments were performed in accordance with the Guide for the Care and Use of Laboratory Animals and approved by the New York State Psychiatric Institute Animal Care and Use Committee.

### X-irradiation

Similar to previous studies [9, 12], 8-week-old mice were anesthetized with ketamine and xylazine (150 mg/kg and 10 mg/kg respectively). NG + mice were untreated. NG− mice were placed in a stereotaxic frame, covered by a lead shield with a 3.22 x 11 mm opening over the hippocampus (interaural 3.00 to 0.00) and placed in a X-RAD 320 biological irradiator (PXI; North Branford, CT). The X-RAD 302 operated at 300kV and 12 mA with a 2-mm AI filter and delivered 2.5-Gy doses per X-ray session. Mice were treated for three sessions, separated by a 2-day interval (day 1, 4 and 7) so NG− mice received a total dose of 7.5-Gy.

### Valgancyclovir Treatment In Gfap-tk Mice

Chow containing VGCV (165 mg/kg) was administered to mice at 8 week until 5 months of age. Mice were on a feeding schedule of chow where they were fed VGCV chow for 5 days and normal chow for 2 days. This feeding schedule was employed to reduce gastrointestinal side effects caused by Tk expression in gut tissues. To assess the effect of VGCV on cell proliferation, tissue from GFAP-Tk^−/−^ (NG + ^TK^) and GFAP-Tk^+/−^ (NG−^TK^) mice were immunolabeled for a marker for cell division Ki-67 and a marker of immature neurons DCX.

### Viral Injections:

To visualize cell bodies projecting to the dorsal dentate gyrus, we used a canine adenovirus, expressing GFP (CAV-GFP), which is taken up by axon terminals and transported to cell bodies [13]. NG + and NG− mice at 5 or 2 months after treatment were used. Mice were anesthetized with a ketamine, xylazine, acepromazine mixture (65mg/kg, 13mg/kg, 1.5mg/kg respectively) and placed into a stereotaxic frame (David Kopf Instruments) with the skull exposed. A 10μl Hamilton syringe with pulled glass pipette was used to infuse 5 x 10^12^ virions of CAV-GFP to the right dorsal hilus (bregma coordinates: anteroposterior – 2.3mm, mediolateral 1.6mm, dorsoventral – 1.6mm) at 0.2 μl/min. Mice were sacrificed 1–4 weeks following surgery.

To visualize cell bodies projecting to the dorsal hippocampus and the ventral hippocampus, we infused CAV-GFP in the dorsal hippocampus and CAV-cherry in the ventral hippocampus. A group of 7-month-old NG + and NG− mice were infused with CAV-GFP in the right dorsal hilus as described above. In addition, the right ventral hilus (bregma coordinates: anteroposterior – 3.2mm, mediolateral 2.3mm, dorsoventral – 4.3mm) was infused with 5 x 10^12^ virions of CAV-mCherry at 0.2 μl/min. Mice were sacrificed 1–4 weeks following surgery.

### Immunohistochemistry

Mice were anesthetized with a ketamine and xylazine mixture (150mg/kg and 10mg/kg respectively) and transcardially perfused with ice cold phosphate-buffered saline (PBS; pH 7.4) followed by 4% paraformaldehyde (PFA) in PBS. Brains were stored in 4% PFA overnight and transferred to 30% sucrose for 48h. Brains were sagittally sectioned at 35 μm and stored in PBS with 0.02% azide. For immunostaining tissue was washed in PBS, blocked with 10% normal donkey serum and incubated in primary antibody overnight at 4°C. The following primary antibodies were used and diluted in 10% normal donkey serum: rabbit Ki67 (1:100 Vector laboratories), goat DCX (1:500; Santa Cruz Biotechnology), chicken GFP (1:500; Abcam), rabbit Living Colors^®^ DsRed (1:1000; Clontech), mCherry (1:250; Clontech), rabbit S100b (1:1000; Abcam), goat GFAP (1:1000; Abcam), guinea pig Parvalbumin (1:1000; Synaptic Systems), S Neurotrace (Life Technologies) served as a counterstain. All fluorescent secondary antibodies were obtained from Jackson ImmunoResearch and diluted 1:200 in PBS.

As outlined in the Allen Reference atlas [14], 20 sagittal sections (S1-S20) of each hemisphere per mouse spaced at 200 μm intervals was analyzed. GFP or Cherry labeled cells in the lateral NDB were counted in sections S13-S15. GFP or Cherry cells in the medial NDB were counted in sections S16-S18. GFP or Cherry cells in the lateral MS were counted in section S20. We did not collect consistent intact samples of section S21 and could not assess the medial MS. For the LC, GFP cells were counted in section S15. LC cells were only detected ipsilateral to the injection. Cherry cells were only detected in the ipsilateral hemisphere. To normalize for viral infection efficiency in each individual mouse, the number of cells in the region of interest (MS, NDB or LC) was divided by the number of cells adjacent to the viral injection site in the dentate gyrus. GFP or Cherry cells were counted in the dentate gyrus, in sections S7 and S8 and averaged. All GFP or Cherry cells in the MS-NDB in sections S13-S20 were assessed for ChAT co-expression and total ChAT cells. DCX and Ki67 were counted unilaterally in 5 sections of dentate gyrus that spanned the septotemporal axis.

### Fluorimetry

Chat-Cre animals were sham or x-irradiated. After 12–14 months, animals were injected with AAV-hEF1α-LS1L-hM3Dq-mCherry. 5 weeks later animals were perfused with 0.1% saline followed by 4% PFA. The mouse brains were serially sectioned using a cryostat into 40 μm sections. 3 atlas matched sections were used for each animal. The sections were immunolabeled with antibodies against mCherry (1:250, Clontech). Images were captured with an SP8 (Leica) confocal microscope using a 10X objective. Acquisition parameters were adjusted to obtain the brightest image for which there was no signal saturation and then maintained identical for all subsequent captures. Hilar fluorescence intensity was measured by manually outlining the dorsal hilus region of interest (ROI) in every section using NIH Fiji software. Signal was normalized to background fluorescence for each capture. Normalized fluorescence was calculated by dividing ROI fluorescence by background fluorescence and reported as arbitrary fluorescence units (AU).

### Imaging And Analysis

Tissue was imaged at 20x on a fluorescent microscope (Olympus IX83). The Allen Brain Atlas was used to define brain regions in sagittal sections. GFP or cherry expressing cells were counted in the medial septum and the diagonal band ipsilateral and contralateral to the injection in sections that transversed the structure. Multilabel high resolution imaging for Fig. 4 was carried out on a Leica SP8 confocal microscope through a HCPLAPO 63X/1.4NA Oil objective with a 3X zoom. The pinhole was set to 1AU. 0.3μm Z-steps were used for acquisition. The final voxel size was 0.12μm x 0.12μm x 0.3μm. Image processing was carried out in NIH Fiji software by applying all adjustments to the entire image. No gamma adjustments were made. 3D reconstruction of astrocyte and cholinergic axons was carried out in the Leica Application Suite X software.

### iDISCO and axonal tracing.

Animals were euthanized by cervical dislocation, decapitated, and brains were rapidly removed and placed on ice. The septohippocampal circuit was microdissected and placed into 4% PFA at 4°C for 2 days. The clearing and staining protocols were performed as described previously [15]. Chicken anti-GFP (AvesLab) was used to label CAV2-GFP and Rabbit anti-RFP (Rockland) was used to label mCherry. Antichicken AlexaFluor 647 and anti-rabbit AlexaFluor568 were used for visualization. Cleared and labeled septohippocampal circuits were imaged using a Leica SP8 Confocal Microscope. Multiple z-stacks were captured using a 10x dry objective, at 1024x1024 resolution, and 2X zoom. The pinhole was set to 1AU. Z-steps were set to 4.283μm resulting in a 0.569μm x 0.569μm x 4.283μm voxel size. Imaris Software (Oxford Instruments) was used to align and stitch z-stacks, trace and reconstruct axonal projections, and render images and video.

### Behavioral Experiments

All behavioral experiments were performed by took place during the light cycle between 9AM and 3PM. Mice with reduced neurogenesis for 2 months were tested in the following order: spontaneous alternation, open field, fear conditioning. Mice with reduced neurogenesis for 5 months were tested in the following order: spontaneous alternation, open field, elevated plus maze, balance beam. Mice without neurogenesis for 4 months were tested for spontaneous alternation. A group of mice without neurogenesis for 2 months and 12 months were tested in spontaneous alternation and used for pharmacological experiments.

### Spontaneous Alternation

To assess spontaneous alternation mice were tested in a closed arm plus maze as described with modifications [16, 17]. The plus maze consisted of four identical arms (25 x 5 x 30 cm) with opaque walls that extended from a center platform (5 x 5 cm) elevated 50 cm from the floor. Testing occurred in a lit (250 lux) room. Mice were placed on the center platform and allowed to explore freely for 12 min. The sequence of arm entries was scored throughout the 12 min. A successful alternation occurred when a mouse made four discrete arm entries on overlapping sets of five consecutive entries. Accordingly, the number of successful alternations is the total number of entries minus four. The spontaneous alternation score is calculated by (successful alternations/total possible alternations) x 100; a score of 44% reflects chance performance.

### Open Field

Mice were placed in a Plexiglas open field (Kinder Scientific SmartFrame 22.1" x 22.1" x 15.83") illuminated by 80–100 lux for thirty minutes. Behavioral measures were automatically recorded by infrared photo beams and analyzed by MotorMonitor software.

### Elevated-plus Maze

The elevated plus maze was performed as described [18], and consisted of a central platform (5 x 5 cm) with two opposing open arms (25 x 5 cm) and two opposing arms enclosed by opaque walls (25 x 5 x 30 cm), elevated 50 cm from the floor. Experiments were conducted in the dark with open arms illuminated (100–120 lux). Mice were placed on the central platform facing a closed arm; the number of entries to each arm and duration in each arm was scored for 5 min by an experienced observer.

### Balance Beam

Beam walking was assessed as described [19]. Mice were given 5 training trials where they traversed a 100 cm long, 1.5 cm diameter circular beam in a lit room (250 lux). 24 hours after training mice traversed the beam once while the number of foot slips and latency to cross the beam were scored.

### Microdialysis

We performed microdialysis to measure baseline and spontaneous alternation induced acetylcholine levels in the dorsal hippocampus as previously described with modifications [16]. NG + and NG− mice aged without neurogenesis for 1.5 months or 4.5 months were implanted bilaterally with microdialysis guide cannulae (Synaptech, S-3000) in the dorsal hippocampus (bregma coordinates: anteroposterior – 2.3mm, mediolateral 1.6mm, dorsoventral – 0.6mm). The cannulae were implanted 1 mm above the dorsal hilus target region as we used a 1 mm membrane to sample from the dorsal hilus. The cannulae were secured to the skull with skull screws and dental cement and mice recovered from surgery for at least 2 weeks.

Microdialysis samples were collected at baseline, during spontaneous alternation and after spontaneous alternation. One group of NG + and NG− mice were tested after 4 months without neurogenesis with a probe in the left hippocampus. Another group of NG + and NG− mice were tested after 5 months without neurogenesis with a probe in the right hippocampus and again after 7 months without neurogenesis with a probe in the left hippocampus.

To begin a trial, a microdialysis probe with a 1 mm membrane (Synaptech, S3010 Synaptech Technology Inc., Marquette, MI) was inserted into the dorsal hilus at the same coordinates as the dorsally infused CAV-GFP (bregma coordinates: anteroposterior – 2.3mm, mediolateral 1.6mm, dorsoventral – 1.6mm) and mice were placed in an opaque holding cage with fresh bedding. The probes were continuously perfused with 100 nM neostigmine bromide (Sigma) in artificial cerebrospinal fluid (aCSF; 128 mM NaCl, 2.5 mM KCl, 1.3 mM CaCl_2_, 2.1 mM MgCl_2_, 0.9 mM NaH_2_PO_4_, 2.0 mM NaHPO_4_, and 1.0 mM glucose at a pH of 7.4) at 1 μl/min. For the first 60 min mice acclimated to the probe and dialysate was not collected. After this period dialysate samples were collected every 6 min. The first 4 baseline samples were collected while the mouse was in the holding cage. After the baseline sampling time mice were placed in the spontaneous alternation task for 12 min while 2 samples were collected. Finally, mice were placed back into the holding cage while 2 post-maze samples were collected.

### HPLC

Dialysate samples were assayed for acetylcholine using HPLC with electrochemical detection (Eicom USA, San Diego, CA). Acetylcholine peaks were quantified by comparison to peak heights of standard solutions and corrected for in vitro recovery of the probe. The system detection limit is reliably 5 femtomole of acetylcholine. Chromatographs obtained every 15 min/sample were analyzed using the software program Envision (provided by Eicom, USA).

### Fiber Photometry

Animals underwent stereotactic surgery 1-week following x-ray irradiation as described above. AAV9-hSyn-ACh3.0 (Vigene Biosciences Inc, Dr. Yulong Li) was injected unilaterally into the left DG (AP −1.96mm, ML −1.26mm, DV −1.98mm from bregma) at a rate of 100nL/min for 5 min. A fiber optic cannula (400μm diameter, 3mm length, Doric Lenses) was lowered 0.05mm above the injection site and was secured with 3 bone screws and dental cement. Mice were allowed 3–4 weeks to recover from surgery and for optimal expression of GRAB-ACh3.0 in the DG. Mice were habituated to being tethered to the fibers for 3 days in their home cage prior to exploring the maze. On the day of the experiment, mice were first placed in a holding box for 15 minutes to habituate from being transported from their housing. Then they were connected to the patch cords and baseline signal was recorded for 10 minutes, before placing the animals in the maze for 12 minutes. The recording was performed using the Neurophotometrics system with 470nm and 415nm LEDs (signal and reference respectively) acquired interleaved by a CMOS camera sensor at 40fps rate. The data was collected using the open-source software Bonsai by selecting a region of interest over the image of the patch cord and calculating the mean pixel value for each time point. Data was analyzed in Matlab (Mathworks) using custom code. Briefly, we fit the signal and reference traces independently to remove their bleaching artifact with a polynomial function. Then, we subtracted the reference to remove any moving artifacts. The signal was normalized to the last 5 minutes of baseline before the animals entered the maze and we used (F-mean(baseline))/sd(baseline) to calculate df/f time series that was used for the remaining calculations. To calculate the rise time (10–90), rise slope (10–90) and rise magnitude (90) upon entering the maze, the signal was smoothed with a bandpass filter (0.02-0.2Hz) and we looked in the first 1 minute in the maze for the local maximum, as well as the 10% and 90% of that value.

### Pharmacology

Scopolamine hydrobromide (Sigma), 5 μg/kg dissolved in 0.9% saline was delivered i.p. 40 min prior to the spontaneous alternation task. Physostigmine hemisulfate (Tocris) 20 μg/kg dissolved in 0.9% saline was delivered i.p. 15 min prior to the spontaneous alternation task or during microdialysis. Clozapine-n-Oxide (NIMH) 10mg/kg was delivered i.p. 15 min prior to the spontaneous alternation task.

### Statistics

All statistics were calculated in GraphPad Prism. All data are presented as the means ± SEM and significance was set at *p* < 0.05. ANOVAs that yielded statistically significant main effects were followed with Bonferroni or Tukey’s *post hoc* tests.

## Results

### Long-term suppression of neurogenesis induces reorganization of cholinergic hilar inputs.

**Figure 1:**
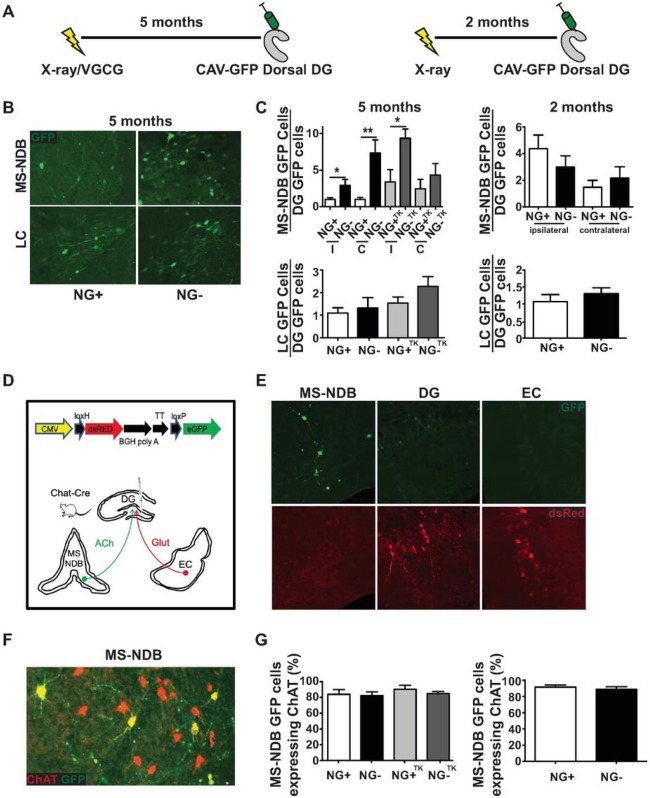
Increased cholinergic hilar inputs in mice living with diminished neurogenesis. (A) 2 month-old mice were exposed to focal hippocampal X-irradiation or VGCV. CAV2-GFP was injected into the dorsal hilus 5 months after X-irradiation or VGCV treatment. CAV2-GFP was also injected into a group of X-irradiated mice after two months. (B) GFP+ cells in the MS-NDB and the LC in NG+ and NG− mice (n=12 per group). (C) NG− mice without neurogenesis for 5 months showed significantly more cells projecting to the dorsal hilus than NG+ mice from the MS-NDB both ipsilateral (I) (t(22)=2.238, p= 0.0357) and contralateral (C; t(22)=3.316, p= 0.0033) to the injection site. NG− mice without neurogenesis for 2 months (n=5) and NG+ mice (n=5) showed similar connectivity from MS-NDB and LC to the dorsal hilus. NG−^TK^ mice with suppressed neurogenesis for 5 months showed significantly more cells projecting to the dorsal hilus than NG+^TK^ mice from the MS-NDB ipsilateral to the injection site (t(7)=2.927, p= 0.0221). (D) Cartoon schematic for CAV2-STOPLIGHT injections into hilus of ChAT-CRE mice. (E) Coronal sections from ChAT-Cre mice injected with CAV2-STOPLIGHT reveal exclusively GFP+ cells in the MS-NDB, and exclusively dsRed+ cells in the entorhinal cortex (EC) and the DG granule cell layer. (F, G) In the MS-NDB of NG+ and NG− animals from B and C, 80-90% of GFP labeled cells overlap with a primary marker of cholinergic cells, acetylcholineserase in 5 and 2 month groups. I= Ipsilateral, C= Contralateral. Bars represent mean ± SEM.

We hypothesized that over time, reduction in neurogenesis would influence connectivity to the hilus, a structure where hippocampal afferents from many brain regions terminate to regulate neurogenesis, granule cell activity, and hippocampal function [20]. To model a reduction in hippocampal neurogenesis across aging, 2-month-old mice were treated with focal hippocampal X-irradiation [9, 12] thereby dramatically and permanently reducing hippocampal neurogenesis (Figure S1). This method is thought to acutely target proliferating cells and also interfere with neural differentiation in the subgranular zone without substantially influencing hippocampal and extra-hippocampal structures [9]. We then used recombinant canine adenovirus (CAV2), which undergoes selective retrograde neuronal transport [13, 21-23], to assess changes in hippocampal inputs over time. We injected CAV2-encoding green fluorescent protein (CAV2-GFP) into the dorsal hilus of NG+ (Sham-irradiated mice with normal neurogenesis) and NG− (X-irradiated mice with diminished neurogenesis) animals after extended periods of reduced neurogenesis (Fig. 1A, S2). We observed GFP + cell bodies in numerous brain regions including the medial septum-nucleus of the diagonal band (MS-NDB) where the number of labeled cells appeared to be different in NG− mice after 5 months of reduced neurogenesis. MS-NDB was also particularly interesting since its hippocampal cholinergic projections are thought to regulate neurogenesis [24, 25], be important for short-term memory [16], and exhibit deleterious changes during aging [26, 27]. Compared to NG+, NG− mice had more GFP +cells in the MS-NDB both ipsilateral and contralateral to the injection site 5 months after irradiation (Fig. 1B,C; S2B-D), indicating that additional MS-NDB neurons were projecting to the hilus in NG− animals. Importantly, there was no difference detected in the number of locally transduced cells (Figure S2A). The increase in the septohippocampal inputs was not detectable in NG− mice with a 2 month-long reduction in neurogenesis (Fig. 1C), indicating that this type of remodeling requires extended periods of living without neurogenesis. The change in connectivity was not detected in another major hilus projection, the locus coeruleus (LC; Fig. 1B,C), indicating that reduction of neurogenesis causes reorganization of some, but not other hilar inputs. We wanted to verify that reorganization of hilar inputs from the MS-NDB observed after 5 months was due to suppression of neurogenesis rather than an off-target effect of X-irradiation. We therefore used a genetic technique to suppress neurogenesis for 5 months in 2-month-old mice and repeated our tracing experiments. New neurons generated in the subgranular zone develop from radial astrocytes that express glial fibrillary acidic protein (GFAP) [11]. We used mice that express herpes simplex virus thymidine kinase (Tk) under control of a GFAP promoter to suppress neurogenesis by administration of the antiviral drug valgancyclovir (VGCV) for 5 months. This approach was validated for targeting dividing stem cells and diminishing neurogenesis while sparing non-stem astrocytes [11]. After 5 months of VGCV treatment we observed suppression of neurogenesis in NG−^TK^ mice (GFAP-Tk+/− mice treated with VGCV for 5 months) compared to NG + ^TK^ (Tk−/− littermates treated with VGCV for 5 months; Figure S1B). We also observed that more cell bodies were labeled in the MS-NDB but not in the LC of NG−^TK^ mice compared to NG + ^TK^ mice (Fig. 1C) echoing the results observed in X-irradiated mice. Together the results demonstrate reorganization of select septohippocampal inputs in animals with diminished neurogenesis.

**Figure 2:**
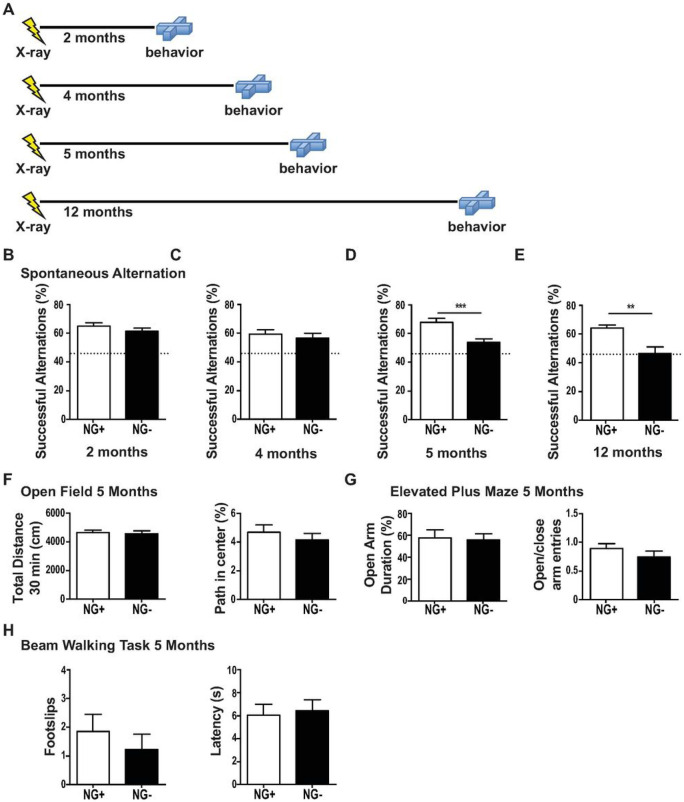
A working memory deficit emerges in mice after a prolonged reduction of adult neurogenesis. (A) Behavior of NG− mice was assessed at 2, 4, 5 and 12 months without neurogenesis and compared to age matched NG+ controls. (B-E) Spontaneous alternation pattern (SAP) in a 4-arm spontaneous alternation task. (B,C) NG− mice after 2 and 4 months without neurogenesis had similar SAP scores of about 60% as NG+ mice (2 months n=15 per group, 4 months NG+ n=12 NG− n=13). (D) However, after 5 months without neurogenesis a deficit emerged in NG− mice where they showed SAP scores around 50% (NG+ n=10, NG− n=14;t(22)=3.835, p= 0.0009). (E) After 12 months without neurogenesis SAP scores declined to chance levels of about 44% (dotted line) NG− mice (NG+ n=7, NG− n=5; t(10)=4.06, p= 0.0023). (F) In the open field, NG− mice (n=11) without neurogenesis for 5 months and NG+ mice (n=8) showed no differences in total distance travelled in 30 min (cm) or the percentage of time spent in the center. (G) In the elevated plus maze NG− mice (n=8) without neurogenesis for 5 months and NG+ mice (n=11) showed similar open arm duration (s) and similar ratios of open to closed arm entries. (H) In a beam walking task, NG− mice (n=6) without neurogenesis for 5 months and NG+ mice (n=6) showed a similar number of total footslips and traversal latency (s). Bars represent mean ± SEM.

The MS-NDB hippocampal projection is comprised of roughly two thirds GABAergic, one third cholinergic and a small proportion of glutamatergic neurons [20, 28, 29]. All three cell types project to the hilus [20, 28, 29]. To determine the identity of CAV2-transduced cells, we turned to CAV2-Stoplight, a CAV2 engineered to induce eGFP expression in transduced cells expressing Cre and dsRed expression in all other transduced cells [30]. CAV2-Stoplight was injected into the hilus of ChAT-Cre animals, which express Cre exclusively in cholinergic neurons [31] (Fig. 1D). Analysis of transduced brains revealed exclusively eGFP + cells in the MS-NDB indicating that only cholinergic MS-NDB neurons were transduced in the hilus (Fig. 1E). In contrast, glutamatergic granule cells of the dentate gyrus and neurons of the entorhinal cortex expressed exclusively dsRed, further illustrating genetic restriction of eGFP expression in our system. Furthermore, immunolabeling brains from NG + and NG− animals revealed that 80–90% of CAV-GFP labeled MS-NDB neurons in NG+, NG−, NG + ^TK^ and NG−^TK^ mice co-expressed ChAT, but not markers for the dominant GABAergic populations residing in the region (Fig. 1F,G, FigS2). Together, the results signify that CAV2 primarily transduces cholinergic hilar inputs. In the autonomic nervous system mature neurons are occasionally thought to convert to a ChAT expressing phenotype [32]. Moreover, some CNS neurons can begin to express new identity markers in adult animals [33]. We therefore also examined if the total number of ChAT + cells was changed in animals living without neurogenesis. We found that the total number of cholinergic neurons in the MS-NDB was not different in NG + and NG− mice or NG + ^TK^ and NG−^TK^ mice (**Figure S3**), suggesting that the increase in labeled cells was due to recruitment of inputs into the region from already existing cholinergic neurons that project elsewhere in NG + mice.

### Ongoing neurogenesis maintains working memory.

Aging and chronic stress reduce neurogenesis [6, 34] while compromising cholinergic function [35, 36] and short-term memory [34]. Therefore, an increase in cholinergic inputs because of neurogenesis ablation seemed surprising. We therefore hypothesized that increased inputs in our models of reducing neurogenesis across aging reflected a natural compensation for an evolving impairment in cholinergic function. We tested NG− mice in a 4-arm spontaneous alternation task [17], which is dependent on an intact cholinergic septohippocampal projection [16]. We found that NG + and NG− mice performed at similarly when neurogenesis was reduced for 2 and 4 months. However, a deficit in short-term memory emerged in NG− mice after 5 months, coinciding with changes in the septohippocampal projection. This deficit remained after 12 months without neurogenesis, when NG− animals performed at chance levels demonstrating a complete failure of working memory in this task (Fig. 2A-E). Thus, a reduction of neurogenesis has a slowly emerging, but profound effect on a cholinergic-dependent working memory task.

Several learning and anxiety tasks have been examined in mice with short-term ablation of hippocampal neurogenesis [37]. We confirmed reports that NG− mice have an impairment in a form of contextual fear conditioning after 8 weeks without neurogenesis, but display unimpaired anxiety-related behavior in the open field or elevated plus maze (Figure S4 and Fig. 2F,G), neither of which depend on cholinergic functioning or neurogenesis. Importantly, NG− animals were not impaired on a beam-walking task (Fig. 2H), which requires an intact cholinergic nucleus basalis projection to the frontoparietal cortex [38]. This demonstrated that the reduction in neurogenesis alters the septohippocampal projection system, but not other cholinergic circuitry.

### Cholinergic dysfunction precedes decreased ACh tone, MS-NDB - hippocampal input reorganization, and memory deficits.

**Figure 3:**
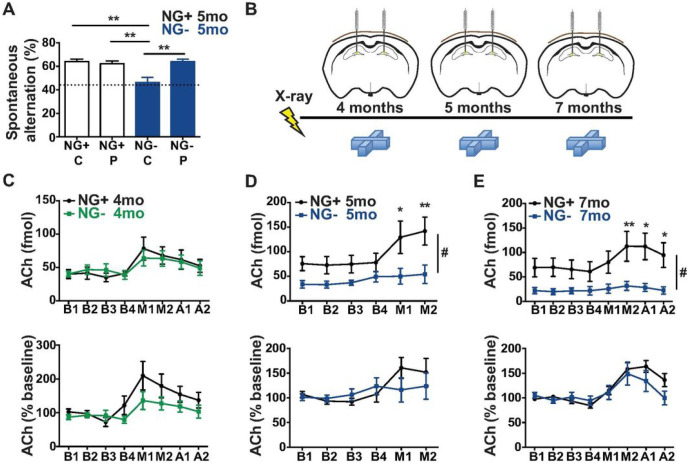
Hippocampal acetylcholine release declines in mice after a prolonged reduction of adult neurogenesis. (A) NG− mice without neurogenesis for 12 months and NG+ mice were administered an acetlycholinesterase inhibitor physostigmine and spontaneous alternation pattern was assessed in a 4-arm spontaneous alternation task. Performance was compared across all groups (NG+ C n= 7, NG− C n=5, NG+ P n=7, NG− P n=5, main effect of group F_326_ = 8.683, p= 0.0007). NG− C mice performed below all groups and at chance. Bars represent mean ± SEM, Tukey’s *post hoc* * p<0.05, ** p<0.01. (B) Mice were treated with X-irradiation or Sham treatment and bilaterally implanted with cannulae. In one group of mice microdialysis measurements were taken from the left dorsal hilus after 4 months without neurogenesis. In a separate group of mice microdialysis measurements were taken from the right dorsal hilus at 5 months and the left dorsal hilus at 7 months without neurogenesis. Microdialysis measurements were taken at baseline (B1-B4), while mice were in the 4-arm spontaneous alternation maze (M1-M2) and after being removed from the maze (A1-A2). (C) After 4 months without neurogenesis NG+ and NG− mice showed similar patterns of acetylcholine release that increased when animals were in the maze (NG+ n= 7, NG− n=8; main effect of time F_7,91_ = 8.504, p< 0.0001). (D) After 5 months without neurogenesis NG− mice showed significantly lower acetylcholine release (n=6 per group; main effect of group F_1,10_ = 5.109, p= 0.0473), main effect of time F_5,50_ = 10.87, p< 0.0001, interaction effect F_5,50_ = 4.198, p= 0.0029, (E) Reduced acetylcholine in NG− mice remained after 7 months with reduced neurogenesis (NG+ n= 5, NG− n=6; main effect of group F_1,9_ = 7.31, p= 0.0242, main effect of time F_7,63_ = 13.44, p< 0.0001, interaction effect F_7,63_ = 6.904, p< 0.0001). (C) Mice without neurogenesis for 4 months demonstrated an increase in acetylcholine above baseline during the spontaneous alternation task. NG− mice showed a slightly attenuated increase in acetylcholine while performing spontaneous alternation compared to NG+ controls (main effect of time F_7,91_ = 8.921, p< 0.0001, main effect of group F_7,91_ =1.874, p=0.1942, time x group interaction F_7,91_ = 1.786, p= 0.0996).b (D) Mice without neurogenesis for 5 months demonstrated an increase in acetylcholine above baseline during the spontaneous alternation task (main effect of time F_5,40_ = 3.695, p= 0.0076) as did animals after 7 months without neurogenesis (main effect of time F_7,63_ = 10,68, p< 0.0001) (E). Bars and points represent mean ± SEM, Bonferroni *post hoc* *p<0.05, ** p<0.01.

Since ablation of hippocampal neurogenesis selectively affected cholinergic inputs from the MS-NDB, we hypothesized that deficits in spontaneous alternation could be rescued in our animals by bolstering the endogenous cholinergic tone in NG− mice. We thus administered physostigmine, a cholinesterase inhibitor, which increases extracellular acetylcholine, to NG− mice and acutely and fully restored the spontaneous alternation deficit even after 12 months without neurogenesis (Fig. 3A) indicating that an acetylcholine deficit was the likely cause of working memory decline in NG− animals. We therefore used awake-behaving microdialysis and HPLC to directly measure hippocampal acetylcholine efflux both at baseline and during spontaneous alternation in NG− mice (Fig. 3B). First, we confirmed that systemic administration of physostigmine increases hippocampal ACh (Figure S5). Next, we observed that only a prolonged reduction in neurogenesis led to reduced acetylcholine in the DG. Four months without neurogenesis resulted in no change in DG acetylcholine levels at baseline or during spontaneous alternation (Fig. 3C). However, after 5 and 7 months without neurogenesis, NG− mice exhibited reduced DG acetylcholine both at baseline and during spontaneous alternation (Fig. 3D,E). Moreover, both NG + and NG− groups at 4, 5 and 7 months mounted an increase in acetylcholine efflux while performing the spontaneous alternation task (Fig. 3C-E), indicating that the cholinergic projection was engaged but the total release was attenuated. Interestingly, the 4-month group had a slightly attenuated acetylcholine increase while performing spontaneous alternation (Fig. 3C) raising a provocative possibility that event-related cholinergic release declines in NG− animals before the anatomic, biochemical, and behavioral phenotypes manifest.

**Figure 4:**
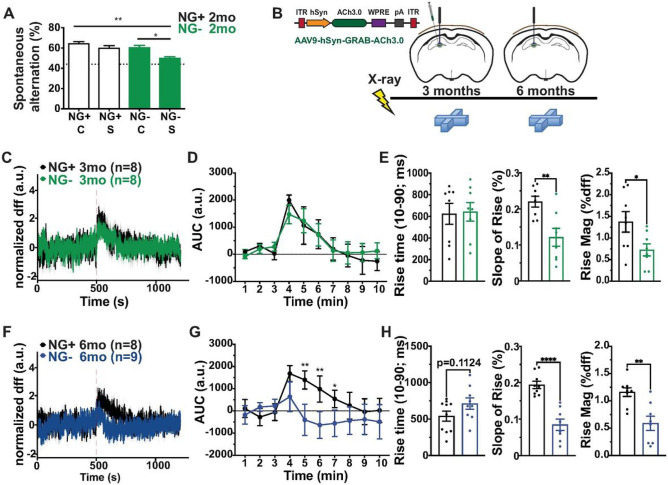
Hippocampal acetylcholine release kinetics are altered in mice after reduction of adult neurogenesis. (A) NG− mice without neurogenesis for 2 months and NG+ mice were administered a muscarinic acetylcholine receptor antagonist scopolamine and spontaneous alternation pattern was assessed in a 4-arm spontaneous alternation task. Performance was compared across all groups (NG+ C n= 15, NG− C n=15, NG+ S n=5 and NG− S n=6, main effect of group F_3,37_ = 4.977, p= 0.0053). The performance of NG− S mice was reduced compared to all groups to near chance levels (dotted line). (B) NG+ and NG− mice were injected with AAV9-hSyn-GRAB-ACh3.0 into the dorsal hilus and implanted with a fiber optic cannula. Fiber photometery was performed during spontaneous alternation testing at 3 and 6 months after neurogenesis ablation. Average signal analyzed across time (C) and in 1-minute bins. (D) revealed a robust increase in ACh signaling after mice entered the maze (broken line) but no differences between NG+ and NG− animals after 3 months of living without neurogenesis (NG+ n=8, NG− n=8; main effect of time F_9,99_=8.844, p<0.0001; main effect of group F_1,11_=0.008527, p=0.9281; time x group interaction F_9,99_=0.4304, p=0.9158; 2-way RM ANOVA and Bonferroni *post hoc*). (E) Task-evoked rise time of baseline acetylcholine was also not different between groups (NG+ n=8, NG− n=8, t(14)=0.1491, p=0.8836). Both the slope and magnitude of rise in response to the maze was significantly lower in NG− mice (slope: NG+ n=8, NG− n=8, t(14)=3.434, p=0.0040; magnitude: NG+ n=8, NG− n=8, t(14)=2.357, p=0.0335; *p<0.05, ** p<0.01). (F,G) After 6 months without neurogenesis, NG− mice exhibited an attenuated task-evoked increase in acetylcholine signaling compared to NG+ animals (NG+ n=9, NG− n=9; main effect of time F_3.505, 45.57_=9.909, p<0.0001, main effect of group F_1,13_=40.18, p<0.0001, time x group interaction F_9,117_=7.776, p<0.0001; 2-way RM ANOVA and Bonferroni *post-hoc*, *p<0.05, ** p<0.01). (H) Task-evoked rise time did not differ between NG− and NG+ mice at 6 months (NG+ n=9, NG− n=9, t(16)=1.429, p=0.1723). Both the slope and magnitude of rise was significantly lower in NG− compared to NG+ mice after 6 months (slope: NG+ n=9, NG− n=8, t(15)=6.031, p<0.0001; magnitude: NG+ n=9, NG− n=8, t(15)=3.841, p=0.0016; **p<0.01, **** p<0.0001). Bars represent mean ± SEM.

We tested this possibility by challenging the cholinergic system in NG− mice after 2 months without neurogenesis with the muscarinic cholinergic antagonist scopolamine, which impairs spontaneous alternation behavior in high doses [39]. As expected, at a normally sub-threshold dose, scopolamine did not affect NG + mice, however it profoundly impaired spontaneous alternation behavior in NG− mice even after 2 months without neurogenesis (Fig. 4A) suggesting that cholinergic dysfunction is present in NG− animals long before behavioral or other changes emerge. To identify the mechanism underlying early sensitivity to scopolamine in NG− animals we used the fluorescent indicator GRAB-ACh3.0, a genetically encoded sensor of ACh activity [40, 41]. NG + and NG− animals were injected in the dorsal hilus with AAV-GRAB-ACh3.0 and implanted with optical fibers enabling direct measures of ACh dynamics by fiber photometry while animals performed the spontaneous alternations task (Fig. 4B). As with microdialysis, with fiber photometry we observed a task-elicited increase in DG ACh (Fig. 4C,D). This increase was attenuated in the NG− group after 6 months without neurogenesis (Fig. 3F,G) when low ACh tone was noted (Fig. 3D,E). More detailed analysis revealed that NG− mice exhibited an attenuated response to maze exposure when ACh demands are greatest (Fig. 4E). The deficit was evidenced by a decreased slope of the signal rise to peak resulting from a decreased rise magnitude while the rise time remained unchanged and was present even after 2 months of living without neurogenesis. The scopolamine and GRAB-ACh3.0 experiments together identified a subtle deficit in ACh function that precedes the slowly emerging anatomic, behavioral, and biochemical consequences of living without neurogenesis.

Together the results indicate that reduction in neurogenesis initially results in a subtle ACh deficit, which is followed by attenuated release of phasic acetylcholine, progressing to a tonic efflux deficit and ultimately functional circuit deficits with compensatory changes in cholinergic innervation. Our findings therefore show that ongoing neurogenesis functions to maintain septohippocampal cholinergic function. It also supports the hypothesis that increased septohippocampal innervation observed in Fig. 1 is compensatory to a primary decline in cholinergic function.

### Ventral cholinergic projection is recruited by the dorsal hilus during rewiring and can be mobilized to rescue working memory deficits in NG− mice.

**Figure 5:**
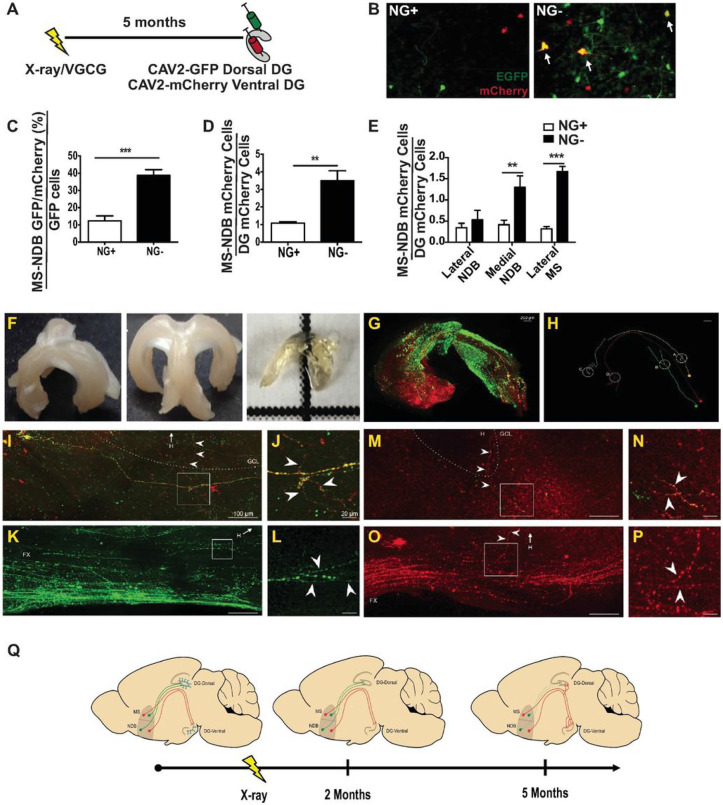
Reorganization of cholinergic septotemporal projection after a prolonged reduction in adult neurogenesis. (A) In NG+ and NG− mice without neurogenesis for 5 months, CAV-GFP was injected into the dorsal hilus and CAV-Cherry was injected into the ventral hilus. (B,C) In NG+ mice (n=5) 10% of GFP+ cells in the MS-NDB showed Cherry labeling compared to 40% in NG− mice (n=4; t(7)=5.985, p= 0.0006). (D) NG− mice (n=5) show a greater number of cells projecting from the MS-NDB to the ventral hilus compared to NG+ mice (n=4, t(7)=4.754, p= 0.0021). (E) The increase in the number of cell bodies in NG− animals is in the medial NDB (t(7)=3.331, p= 0.0126) and lateral MS (t(7)=11.14, p<0.0001). Bars represent mean ± SEM. * p<0.05, ** p<0.0. Mapping hilar input reorganization in NG− mice. (F) Septohippocampal circuit was microdissected from NG− mice, immunolabeled, and subjected to iDISCO clearing. (G) 3-Dimensional reconstruction of confocal microphotographs through a unilateral septohippocampal projection. The brains were immunolabeled for mCherry, which was injected into the ventral DG and GFP, which was injected into the dorsal DG. Note the three neuronal populations 1) solely innervating the dorsal DG (green), 2) solely innervating the ventral DG (red), and 3) innervating both dorsal and ventral DG (yellow). An mCherry+, GFP+, and double+ axon was traced and all three were overlayed onto the reconstruction. (H) Axonal tracings from a MS-NDB neurons projecting to the dorsal DG (Green), ventral DG (Red), and both (Yellow). Filled circles represent cell bodies of origin. Open white circles represent axonal branching points in the fornix as exhibited in panels D-K. Dotted red line indicates region of discontinuity. (I) An mCherry+GFP+ axon forms branches in the fornix that traverse the molecular layer to innervate the dorsal hilus (arrows). (J) Boxed region magnified. (K) The same mCherry+GFP+ axon forms branches in the ventral DG (arrows). (L) Boxed region magnified. (M) A GFP+ axon in the fornix forms branches that traverse the granule cell layer to innervate the dorsal hilus. (N) Boxed region magnified. (O) An mCherry+ axon in the fornix forms branches that traverse the granule cell layer to innervate the ventral hilus. (P) Boxed region magnified. Note Abbreviations: GCL, granule cell layer; Fx, fornix; H, hilus.(Q) Structural and functional reorganization of the septohippocampal circuit in NG− mice. NG+ mice show acetylcholine release that supports working memory and cholinergic afferent organization within the septohippocampal projection. NG− mice without neurogenesis for 2 months show an emerging deficit in acetylcholine release in the hippocampus but maintain cholinergic afferent organization within the septohippocampal projection. NG− mice without neurogenesis for 5 months show significant reductions in hippocampal acetylcholine release and rewiring of cholinergic septohippocampal inputs with septal neurons that normally project to the ventral hilus innervating the dorsal hilus and increased innervation of the ventral hilus.

Normally, the septohippocampal projection has a highly organized topography. In rats, MS-NDB neurons project to either the dorsal or ventral hilus, but not to both [42, 43], though this has not been previously established in mice. We hypothesized that the increase in dorsally projecting MS-NDB cells in NG− mice after 5 months results from recruitment of cholinergic fibers of passage that normally traverse the dorsal to innervate the ventral DG. We first analyzed the topography of MS-NDB cells projecting to the dorsal hilus in NG + and NG− mice. We found that the increase in labeled cells in NG− mice after 5 months with reduced neurogenesis originated from structures that normally project to the ventral hippocampus, the medial NDB and lateral aspect of the MS in rats (Figure S6). These abnormal projections suggested that aging with reduced neurogenesis results in reorganization within the septohippocampal projection so that neurons that normally innervate the ventral DG, innervate both dorsal and ventral DG. We tested this possibility directly by injecting CAV2-GFP into the dorsal hilus and a red reporter CAV2 (CAV2-mCherry) into the ventral hilus in NG + and NG− mice after 5 months of reduced neurogenesis (Fig. 5A). We found that in NG + mice, as in rats, dorsally projecting (GFP-labeled) cells were mostly distinct from ventrally projecting (mCherry-labeled) cells (Fig. 5B). However, in NG− mice ≈ 40% of cells with terminals in the dorsal hilus also had terminals in the ventral hilus (GFP and Cherry labeled; Fig. 5B,C). Importantly, there was no overlap between the two injections due to direct viral spread (Figure S8). We then directly imaged the reorganized neurons in NG− mice by microdissecting the septo-hippocampal projection from animals injected with CAV2-GFP dorsally and CAV2mCherry ventrally and subjected the preparation to the iDISCO staining and clearing protocol (Fig. 5F). We then traced single and double labeled axonal trees from the medial septum along the fornix, to the ventral hilus (Fig. 5G) and directly observed branches which branched off from an mCherry + GFP + axon in the fornix and traversed the dorsal or ventral hippocampi to innervate their DG and hilar targets (Fig. 5F-P Supplemental Movie 1). Together, the results indicate a reorganization of the septohippocampal projection in NG− mice where ventrally projecting MS-NDB cholinergic neurons also develop dorsal projections. Moreover, NG− mice had an increased number of mCherry labeled cells in the MS-NDB, specifically in the ventrally projecting medial NDB and lateral MS compared to NG + mice (Fig. 5D,E) indicating that ventrally projecting MS-NDB cholinergic neurons also demonstrated reorganization within the ventral hippocampus.

Therefore, aging without neurogenesis results in increased innervation, albeit dysfunctional, in both the ventral and the dorsal hippocampus by normally ventrally projecting MS-NDB neurons.

**Figure 6:**
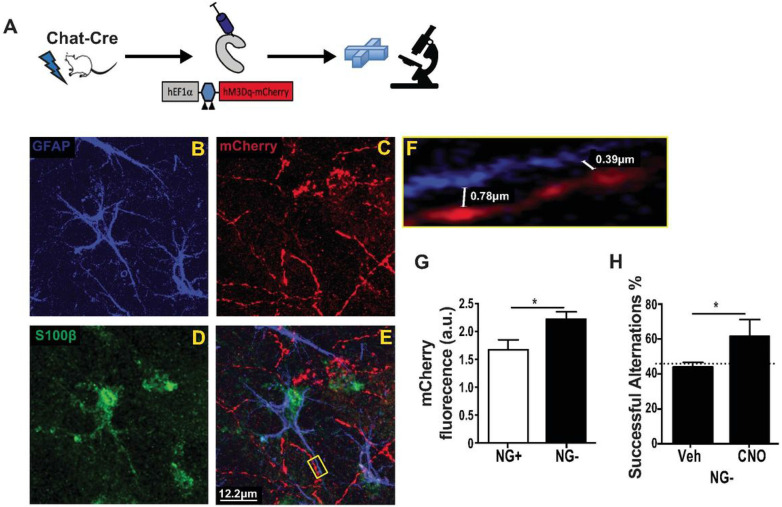
Reorganization of cholinergic septotemporal projection after a prolonged reduction in adult neurogenesis. (A) NG+ and NG− mice were injected with an HSV encoding a cre-dependent form of the activated DREADD hM3Dq and mCherry. Spontaneous alternation was tested with and without CNO and brains were analyzed for hilar mCherry fluorescence and as well as localization and morphology of mCherry+ axons. (B-E) Representative confocal micrograph labeled for markers of Astrocytes (GFAP and S100b) and cholinergic axons (mCherry). Note the proximity of axon segments with ample varicosities surrounding astrocytes. (F) A cutout from (E) demonstrates cholinergic axonal varicosities within 400-800nM from an astrocyte process. (G) Total hilar mCherry fluorescence was greater in NG− (n=6) animals compared to NG+ (n=5) controls (t(9)=2.493, p=0.0171. (H) NG− Mice with DG projecting hM3Dq-expressing cholinergic neurons performed better at the spontaneous alternation task when they received CNO injection prior to testing compared to vehicle control (t(5)=2.134, p=0.043; n=6,6).

We next wanted to directly assess the newly formed cholinergic inputs at their hilar projection site. Engineered Herpes Simplex Viruses (HSVs) are another mainstay tool for retrograde tracing [44, 45] providing opportunity for confirmation of our results obtained with CAV2. We injected an HSV encoding a Cre-dependent form of mCherry-labeled hM3Dq DREADD into the hilus of NG + and NG− ChAT-Cre mice. This approach allowed for mCherry and hM3Dq expression selectively in cholinergic neurons projecting to the hilus and thus both structural and functional analyses of MS-NDB cholinergic neurons (Fig. 6A). Quantitative fluorimetry revealed more mCherry fluorescence in the dorsal hilus of NG + animals (Fig. 5O) indicating more robust cholinergic innervation there. MS-NDB cholinergic neurons are thought to synapse onto DG granule cells [42, 46] including young, adult-born neurons [47, 48]. However, much of the cholinergic signaling in the DG is thought to occur via non point-to-point transmission [46]. Moreover, our intervention (ablating neurogenesis) chronically depleted synaptic partners for cholinergic neurons. We thus examined whether increased cholinergic hilar innervation was anatomically positioned for volume conduction. Recent reports suggest that astrocytes are targets for cholinergic signaling in the hilus and that they are necessary for cholinergic modulation of DG activity. Indeed, we observed varicose mCherry + axon segments in close proximity to hilar astrocytes (Fig. 6B-F, Supplemental Movie 2). High resolution confocal images revealed mCherry + axonal varicosities a fraction of a micron within GFAP + S100b + astrocytic processes (Fig. 4K) demonstrating cholinergic axons well positioned for astrocyte signaling. Interestingly mCherry + axons were rarely observed near parvalbumin (PV)-expressing interneurons, which are thought to be downstream of hilar astrocytes in control of cholinergic modulation of dentate activity [49]. Out of 166 cells examined within 1μm proximity to mCherry + axons, 159 were GFAP + astrocytes and 7 were PV + interneurons. Finally, the distribution of mCherry-expressing hilar axons grossly appeared similar between NG + and NG− mice. Together the data suggest that increased cholinergic innervation is appropriately positioned, but functionally deficient to support working memory. Accordingly, systemic administration of CNO, but not vehicle, rescued working memory deficits in NG− mice expressing hM3Dq in cholinergic neurons (Fig. 6H).

## Discussion

Our findings demonstrate a critical role for ongoing neurogenesis over extended periods of time in the maintenance of cholinergic hippocampal inputs and non-reinforced working memory. This represents a remarkably slow form of plasticity emerging over months. A temporal scale of such a magnitude corresponds to cumulative changes observed during chronic disease and aging. Accordingly, hippocampal neurogenesis is reliably reduced during chronic psychosocial stress, aging, and in Alzheimer’s disease [8, 50]. Here, we have observed that an experimental chronic reduction in neurogenesis is sufficient to result in systems level hippocampal dysfunction over time.

The earliest evidence of subthreshold cholinergic deficits in our study corresponds to time points when numerous, more subtle behavioral deficits associated with ablation of neurogenesis have been described [5, 7, 37]. Our findings therefore raise a possibility that mild cholinergic dysfunction may underlie behavioral deficits normally attributed to the ablation of young neurons. Studies using physostigmine could be used to address this possibility.

Ultimately, a profound degradation of DG-dependent working memory emerged in NG− mice along with a dramatic remodeling of septohippocampal cholinergic DG inputs. Septal cholinergic neurons were previously demonstrated to undergo axonal sprouting in the absence of synaptic targets [51] and temporary remodeling while animals are housed in enriched environments [52]. Our findings indicate that elimination of adult-born neurons results in compensatory rewiring of the septohippocampal projection by recruitment of ventrally projecting axons for dual (dorsal-ventral) innervation. Thus, young dentate granule neurons appear to maintain the integrity of the septohippocampal circuit over the lifespan. We hypothesize that hippocampal neurogenesis serves as a functional target for cholinergic septohippocampal neurons, and in their absence cholinergic cells initially show a reduction in the ability to mount cholinergic responses and then reorganize their innervation as a compensatory mechanism (Fig. 6A).

The progressive nature of behavioral and anatomic changes presented here is reminiscent of neurodegenerative changes observed during age-related cognitive decline and in Alzheimer’s disease, where decreased cholinergic innervation was previously linked to fewer neural stem cells [36]. Hence, neurogenesis may constitute a target for stabilizing the septohippocampal circuit and consequently working memory during normal aging and in disease states. In turn, the described mouse preparation demonstrating slow progressing loss of circuit function can serve as a model for developing pharmacological strategies to acutely treat behavioral consequences. More broadly, given the susceptibility of adult neurogenesis to the effects of chronic stress and aging, it is intriguing to speculate that the adult connectome is more malleable by experiences than currently appreciated. Our results raise the possibility that targeting distinct populations of cells constitutes a viable strategy for rewiring of long-range connectivity in the brain.
